# Severe Hyponatremia in the Emergency Department Incidence of Cerebral Edema and Risk of Osmotic Demyelination Syndrome

**DOI:** 10.1111/acem.70158

**Published:** 2025-10-09

**Authors:** Volker Burst, Ramon Rabii, Julian Peto‐Madew, Thorsten Persigehl, Stefan Haneder, Matthias Johannes Hackl, Christoph Hüser, Moritz Trappe, Sadrija Cukoski, Kathrin Möllenhoff, Victor Suárez

**Affiliations:** ^1^ Emergency Department, Faculty of Medicine and University Hospital Cologne University of Cologne Cologne Germany; ^2^ Department II of Internal Medicine and Center for Molecular Medicine Cologne, Faculty of Medicine and University Hospital Cologne University of Cologne Cologne Germany; ^3^ Department of Diagnostic and Interventional Radiology University of Cologne Cologne Germany; ^4^ Institute of Medical Statistics and Computational Biology (IMSB), Faculty of Medicine University of Cologne Cologne Germany

## Abstract

**Study Objective:**

Treatment strategies in severe hyponatremia aim at rapid sodium correction to prevent or treat cerebral edema but limit sodium rise to prevent osmotic demyelination syndrome (ODS). The true risk of edema or ODS in ED patients is unknown.

**Methods:**

We performed a retrospective study of patients admitted to the ED of a tertiary hospital from January 2013 to December 2018 with plasma sodium ≤ 125 mmol/L. The rate of cerebral edema at presentation and the rate of ODS that developed during the stay were determined based on imaging studies and clinical evaluation. Secondary analyses looked at the association between overly rapid sodium correction (> 8 mmol/L) at 24 h, ODS risk, mortality, and length of stay.

**Results:**

The primary analysis group comprised 852 patients; 318 (37%) of these presented with severe symptoms. Four patients (0.5%) with cerebral edema and 11 patients (1.3%) with ODS were detected. Alcoholism, liver disease, and malnutrition were identified as risk factors for ODS. While overly rapid correction showed no association with ODS in the primary analysis group, it became the predominant risk factor in a reduced dataset with a more accurate estimate of 24‐h sodium correction. Correction rate had no impact on mortality or length of stay.

**Conclusions:**

Given the low rate of cerebral edema even in severely symptomatic patients, aggressive treatment may not be necessary in most cases. The risk to develop ODS seems to be higher than the risk of brain edema. Since we found no beneficial impact of a liberal correction strategy, current treatment limits should stay in place.

## 1 Introduction

### 1.1 Background

Hyponatremia constitutes a frequent cause for presentation to the emergency department (ED) and can pose an immediate vital threat to the patient. Severe reduction of plasma sodium levels and, more importantly, acute development of hyponatremia (i.e., in less than 48 h) can lead to cerebral edema followed by herniation and death [1]. Since the time course of development is usually not known, rapid treatment using intravenous hypertonic solutions is widely recommended if severe neurological symptoms are present, with the rationale being that these symptoms are indicative of cerebral edema [2, 3]. However, severe symptoms can also be found in patients with chronic hyponatremia (i.e., development in > 48 h), which are not at risk for developing cerebral edema due to adaptive countermeasures in the brain [4, 5, 6]. Although severe symptoms are less frequently found in chronic than in acute hyponatremia [7], a higher incidence of chronic hyponatremia may lead to a larger overall number of severely symptomatic patients with chronic hyponatremia. In consequence, many patients with hyponatremia presenting to the ED might receive disproportionate treatment without vital indication. At the same time, such highly active therapy is associated with a significantly increased risk for an overly rapid correction of sodium, which has been linked to the occurrence of the osmotic demyelination syndrome (ODS) [8]. Therefore, current US recommendations limit the sodium increase to a maximum of 8 and 10–12 mmol/L in the first 24 h in patients with high or low‐to‐moderate ODS risk, respectively [2]. Similarly, the 2014 European Best Practice Guideline (ECPG) recommends a limit of 10 mmol/L in each 24 h‐period [3]. Both sodium correction goals as well as limits are difficult to accomplish [9]. Recent publications have questioned the importance of overly rapid sodium correction with regard to ODS and advocate a more liberal treatment strategy [10, 11, 12]. On the other hand, it is unknown which patients are really at risk of developing cerebral edema and therefore require immediate therapy. In order to refine guidance on the management of severe hyponatremia, a better understanding of the proportions and characteristics of patients at risk for cerebral edema on one side or ODS on the other is needed.

### 1.2 Goals of This Investigation

The primary objective of this study was to determine the proportion of cases with cerebral edema at presentation as well as the proportion of ODS cases that developed during the hospital stay in the same cohort of patients with severe hyponatremia. A secondary objective was a more detailed characterization of these subsets.

## 2 Methods

### 2.1 Study Design and Setting

We conducted a retrospective cohort study of all patients who presented to the ED of the University Hospital of Cologne, Germany, with severe hyponatremia, defined as a plasma sodium < 125 mmol/L, between January 1, 2013 and December 31, 2018. Our facility is a hospital‐based ED with approximately 44,000 patient visits annually. The University's Institutional Review Board approved the study (22‐1373‐retro). The methodology employed in this work is in compliance with current recommendations on the conduct of chart reviews [13] and STROBE guidelines [14]. The study was registered at www.clinicaltrials.gov (NCT06781710).

### 2.2 Selection of Participants

We included patients with age ≥ 18 years and a plasma sodium concentration ≤ 125 mmol/L in the initial blood sample obtained upon admission to the ED. Patients were excluded if they had an initial blood glucose level > 300 mg/dL or if follow‐up sodium measurements within the 48 h after admission were unavailable (except for those who died in the ED).

### 2.3 Measurements

Eligible patients were identified through a search of the electronic database maintained by the hospital‐based clinical laboratory. For each patient, we were provided with the following parameters obtained at the initial presentation: sodium, potassium, chloride, phosphate, glucose, creatinine, urea, uric acid, thyroid‐stimulating hormone, hemoglobin, and—if available—serum osmolality, urine osmolality, and urinary sodium within the first 24 h after admission. Furthermore, all sodium measurements obtained during the entire hospital stay by either indirect (Cobas c702, Roche Diagnostics, Switzerland) or direct potentiometry (ABL800Flex, Radiometer, Germany) were recorded and pooled to enhance data density for further evaluation of sodium evolution. The following information was obtained from the hospital's electronic medical record system: age, gender, length of hospital stay (LOS), death, transferal to intensive care unit (ICU) from ED. We then manually performed a full chart review and abstracted the following data points from the ED documentation: etiology of and symptoms attributable to hyponatremia (see specifications below), vital signs, volume status (hypo‐, eu‐, hypervolemic), history of liver disease, malnutrition or alcoholism, pre‐existing medication with known frequent associationss with hyponatremia, and treatment in the ED with a potential effect on sodium levels (isotonic or hypertonic IV fluids, tolvaptan, fluid restriction, loop diuretic, drug withdrawal). In addition, we manually reviewed the entire inpatient records to determine whether signs and symptoms suggestive of osmotic demyelination syndrome (ODS) [8, 15, 16, 17, 18, 19] occurred at any point during the stay (see Appendix S1). Finally, all cerebral computed tomography (CT) and magnetic resonance imaging (MRI) studies performed during the hospital stay and up to 3 months after ED presentation were identified. The search was conducted using both the hospital's medical record system and the Radiology Department's picture archiving and communication system (PACS). Data retrieval was performed by an IT specialist from the Radiology Department.

Data abstraction was performed by two final‐year medical students (R.R. and J.P.‐M.) who were trained by the principal investigators (V.B., V.S., both board‐certified emergency physicians and nephrologists). Two nephrology residents (M.T. and S.C.) served as direct contact persons during data abstraction. A data dictionary (Appendix S2) and a pre‐specified electronic data entry form were provided to the abstractors. They independently analyzed the entire patient set. To ensure data quality and consistency, 15 patient cases were analyzed by the abstractors together with the principal investigators and residents. Periodic meetings were held with the abstractors to resolve uncertainties, and random samples were reviewed to monitor data accuracy, demonstrating a high agreement between the abstractors. At the end of the data collection, all inconsistencies in the clinical data entries were resolved by M.T., S.C., or V.B., V.S. Several meetings were held with the principal investigators and two additional board‐certified emergency physicians (M.J.H., C.H., both otherwise not involved in the data abstraction)—to assess the potential presence of cerebral edema in severely symptomatic patients without available CT/MRI studies (e.g., patients deceased before imaging). Likewise, all patients with symptoms suggestive of ODS were discussed in this group. A diagnosis of ODS was accepted if at least three of the four reviewers independently reached this conclusion after reviewing all available information. Imaging studies were re‐evaluated for either the presence of cerebral edema or signs of demyelination (see specifications below) independently by two board‐certified radiologists (T.P. and S.H.). Both specialists were blinded with regard to individual clinical features and the original report of radiological findings. In case of discrepancies, the case was discussed and re‐evaluated by a third radiologist.

### 2.4 Specifications

#### 2.4.1 Symptoms

In accordance with the ECPG [3], patients were considered severely symptomatic if one of the following symptoms during the past 24 h was reported upon admission: coma (Glasgow Coma Scale [GCS] ≤ 8), seizures, obtundation (somnolence, altered consciousness, confusion), or vomiting. Based on general perception, we graded the symptoms in terms of severity in the above order, with coma representing the most severe condition. Since hypoxia—induced by either respiratory arrest or neurogenic pulmonary edema—has been suggested as a contributing factor (and mentioned by the ECPG as a severe symptom), all patients who either presented with pulmonary distress to the ED (using the key search terms *shortness of breath*, *dyspnea*, *pulmonary edema*, *hypoxia*, *hypoxemia*), showed an SpO_2_ < 90%, had radiological findings of pulmonary edema, or had been intubated prior to admission because of hypoxia were identified. However, since this work focuses on the cerebral damage caused by hyponatremia and its treatment, we have refrained from analyzing pulmonary injury as an outcome parameter and instead provide only the incidences.

#### 2.4.2 Radiological Diagnosis of Cerebral Edema and ODS

Hyponatremia‐induced cerebral edema was defined as diffuse, global edema, with or without signs of transtentorial and/or tonsillar herniation and absence of other global or focal lesions on emergency imaging [20]. Emergency imaging included CT or MRI studies conducted in the ED within the first 2 h after detection of hyponatremia in patients who had either not yet received treatment for hyponatremia or had a sodium rise of ≤ 2 mmol/L. Typical features of demyelination in MRI are hyperintense areas in T2‐weighted and T2 fluid‐attenuated inversion recovery (FLAIR) sequences or hyperdense areas in CT‐scans [19]. Radiological findings of ODS comprise symmetrical demyelination in the central part of the pons (i.e., central pontine myelinolysis) with or without extrapontine demyelination [21, 22] or atypically shaped or located, asymmetrical or multifocal lesions in the pons and/or extrapontine. All imaging studies that had been conducted during the stay and—to account for the frequently delayed appearance of radiological signs—over a period of 3 months from admission were evaluated.

#### 2.4.3 Clinical Diagnosis of Cerebral Edema and ODS

The diagnosis of hyponatremia‐induced cerebral edema was accepted if an alternative explanation for death was lacking and/or one of the following was present: edema in delayed imaging studies while hyponatremia persisted, death of initially comatose patients without intermittent neurological improvement, or death following deterioration of neurological symptoms in initially non‐comatose patients with persisting hyponatremia. The diagnosis of ODS was deemed possible if any of the pre‐defined signs and symptoms had been detected and no other explanation was evident or more likely.

In summary, hyponatremia‐induced cerebral edema was considered “confirmed” if the diagnosis was made based on radiological or clinical re‐evaluation. ODS was considered “confirmed” in patients with clinical features and radiological findings of demyelination. ODS was considered “possible” in patients with clinical but no radiological features (or who had not received imaging).

#### 2.4.4 Sodium Evolution

Overly rapid correction was defined as a sodium increase of > 8 mmol/L at 24 h calculated as sodium at 24 h after initial sodium measurement minus initial sodium. The 24 h‐sodium value was extrapolated using the formula proposed by George et al. [23]. Sensitivity analysis was performed using a reduced dataset comprising only patients that had sodium results available in the timeframe between 18 and 30 h, yielding a more accurate estimate for the true 24 h‐sodium. In total, 475 patients qualified for this latter sub‐analysis: 208 patients had sodium readings only between 18 and 24 h, 195 patients had readings between 18 and 24 h as well as between 24 and 30 h, and 72 patients had readings only between 24 and 30 h. The mean time difference from the 24 h mark was 2.24 h (SD 1.65 h).

### 2.5 Study Outcomes

The two major outcomes were the event rate for confirmed hyponatremia‐induced cerebral edema on admission and the event rate for confirmed ODS during hospitalization. A secondary objective was to characterize the above‐mentioned cohorts with respect to clinical and laboratory features. This also included evaluation of overly rapid correction of sodium levels and its impact on ODS risk.

### 2.6 Statistical Analysis

For the descriptive analysis, numerical variables are displayed as medians (interquartile range [IQR]), categorical and dichotomous variables are given as frequencies and proportions (%). To further delineate the impact of known risk factors on the development of ODS, overly rapid sodium correction, mortality, and length of stay, we performed logistic or linear regression analyses; independent variables were selected a priori according to existing evidence. To account for low event rates, Firth's penalization was applied as appropriate [24]. Data preparation, processing, as well as all analyses were performed using R (R Core Team, R version 4.2.0 [2022‐04‐22]). *p*‐values < 0.05 were considered significant.

## 3 Results

### 3.1 Characteristics of Study Cohort

**FIGURE 1:**
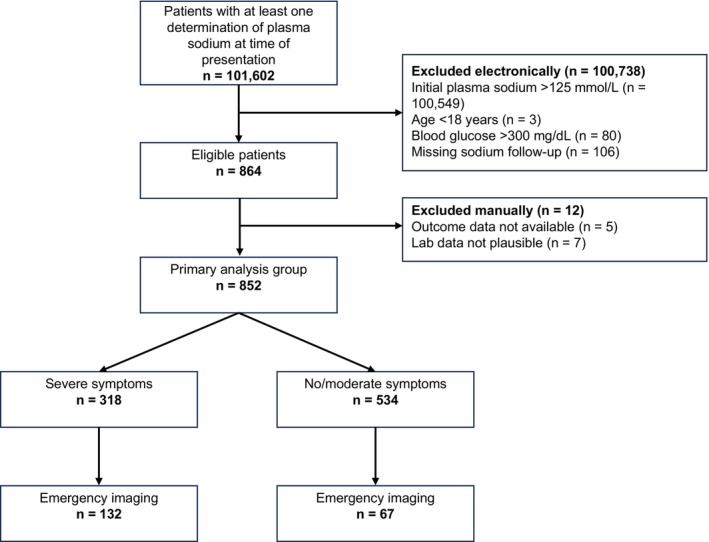
Patient flow diagram.

Of 101,602 patients who presented to the ED in the study period and received laboratory work‐up, 1101 patients had an initial plasma sodium ≤ 125 mmol/L and 852 patients were included in this analysis (Figure 1). 412 (48.4%) of this primary analysis group were men; the median age was 67 years. 203 patients (23.8%) were transferred to an intensive care unit and 93 (10.9%) died during the hospital stay.

**FIGURE 2:**
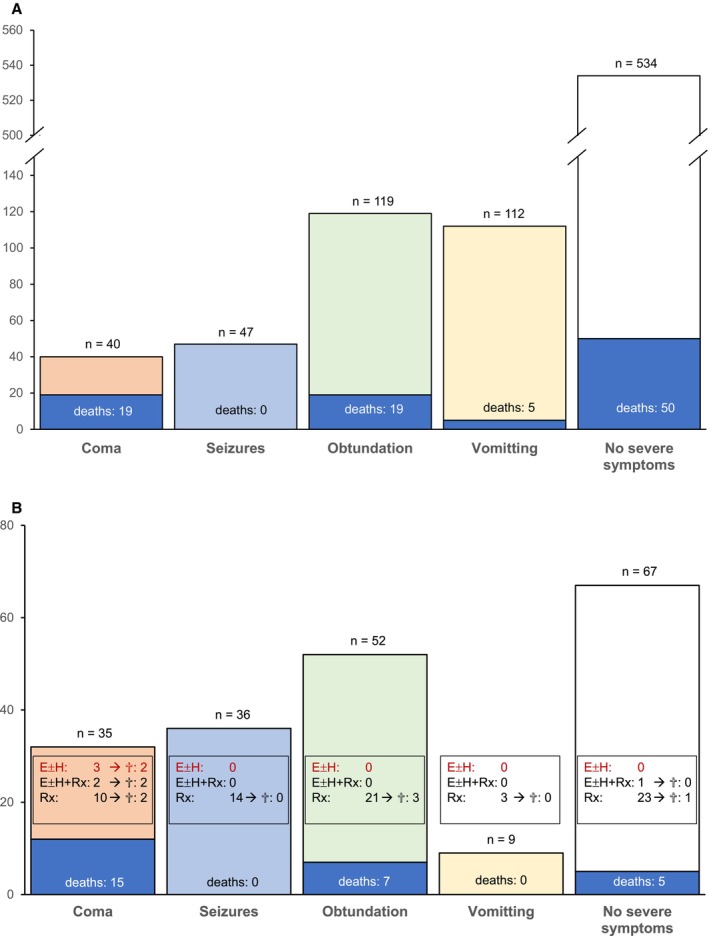
(A) Primary analysis group. Patients with severe hyponatremia grouped according to the most severe symptom presented on admission (coma > seizures > obtundation > vomiting > no severe symptoms) and mortality. (B) Patients of primary analysis group that received emergency imaging of the brain. Patients with severe hyponatremia grouped according to the most severe symptom presented on admission (coma > seizures > obtundation > vomiting > no severe symptoms) and mortality. Boxes: E ± H, global cerebral edema with or without herniation representing true hyponatremia‐induced cerebral edema; E ± H + Rx, global cerebral edema with or without herniation and other focal or global pathologies; Rx, focal or global pathologies other than E or H; ^†^Died.

In total, severe symptoms were reported in 318 patients (37%); 40 patients (4.7%) presented with coma, 54 (6.3%) with seizures, 152 (17.8%) with obtundation, and 152 (17.8%) with vomiting. A total of 32 cases with documented pulmonary distress, hypoxia, or tracheal intubation due to hypoxia were identified; 25 of these patients also presented with severe neurological symptoms. After reviewing all available data, only two cases of respiratory arrest could plausibly be attributed to hyponatremia, as no alternative cause was identified—both patients were initially comatose, deteriorated rapidly into cardiac arrest, and died without undergoing imaging. Three additional patients were diagnosed with confirmed or suspected pulmonary edema; however, two showed signs of acute decompensated heart failure and one had pneumonia. Thus, hyponatremia‐induced neurogenic pulmonary edema appears highly unlikely in our cohort. No apparent or only moderate symptoms were reported for 534 patients (63%). Forty‐three patients (13.5%) with severe symptoms and 50 patients (9.4%) in the group without severe symptoms died. With 19 cases (47.5%), the highest rate of mortality was found in the subgroup of patients that had been admitted with coma. Overly rapid sodium correction occurred in 341 patients (40%), the majority of which received 5% dextrose; only two patients were treated with DDAVP: one received 3 × 2 μg intravenously over 12 h, and the other received a single 40 μg dose via nasal spray. A detailed overview of patient characteristics and demographics is given in Table 1. A bar chart for a better appreciation of the overlap of symptoms and associated mortality is shown in Figure 2A. Additional information on the rate of symptoms and overcorrection at individual sodium levels can be found in Figures S1 and S2, respectively.

**TABLE 1:** Patient characteristics, all patients and patients with severe neurological symptoms.

	Primary analysis group	*N*	Subgroup with severe symptoms	*N*
Male, *n* (%)	412 (48.4)	852	141 (44.3)	318
Age, years, median (IQR)	67 (57, 77)	852	68 (56, 77)	318
Length of stay, days, median (IQR)	6 (2, 13)	852	6 (3, 12)	318
MAP, mmHg, median (IQR)	93.7 (81.7, 107.1)	852	94.7 (79.8, 108)	258
Heart rate, bpm, median (IQR)	80 (70, 95)	852	82 (70, 92)	264
Transferred to ICU, *n* (%)	203 (23.8)	852	116 (36.5)	318
Died, *n* (%)	93 (10.9)	852	43 (13.5)	318
120 mmol/L < [Na^+^] ≤ 125 mmol/L, *n* (%)	498 (58.5)	852	150 (47.2)	318
115 mmol/L < [Na^+^] ≤ 120 mmol/L, *n* (%)	230 (27)	852	95 (29.9)	318
[Na^+^] ≤ 115 mmol/L, *n* (%)	124 (14.6)	852	73 (23)	318
Overcorrection > 8 mmol/L/24 h, *n* (%)	341 (40)	852	158 (49.6)	318
Overcorrection > 10 mmol/L/24 h, *n* (%)	243 (28.5)	852	118 (37)	318
HN etiology				
SIAD, *n* (%)	312 (36.6)	852	125 (39.3)	318
Hypovolemic HN, *n* (%)	230 (27)	852	93 (29.2)	318
Hypervolemic HN, *n* (%)	190 (22.3)	852	49 (15.4)	318
Thiazide‐associated HN, *n* (%)	83 (9.7)	852	36 (11.3)	318
Kidney disease, *n* (%)	16 (1.9)	852	3 (0.9)	318
Water intoxication,^a^ *n* (%)	21 (2.5)	852	12 (3.8)	318
Symptoms, *n* (%)				
Severe neurological symptoms	318 (37.3)	852	—	318
Coma	40 (4.7)	852	40 (12.6)	318
Seizures	54 (6.3)	852	54 (17)	318
Obtundation	152 (17.8)	852	152 (47.8)	318
Vomiting	152 (17.8)	852	152 (47.8)	318
No/moderate neurological symptoms	534 (62.7)	852	—	318
Pulmonary distress	32 (3.8)	852	25 (7.9)	318
Medication with possible association with HN, *n* (%)				
NSAIDs	225 (26.4)	852	73 (23)	318
PPIs	338 (39.7)	852	121 (38.1)	318
Antipsychotic agents	45 (5.3)	852	22 (6.9)	318
Anticonvulsant agents	85 (10)	852	35 (11)	318
Antidepressant agents	122 (14.3)	852	49 (15.4)	318
ACEi/ARB	337 (39.6)	852	106 (33.3)	318
Loop diuretics	259 (30.4)	852	77 (24.2)	318
Thiazides	195 (22.9)	852	65 (20.4)	318
Opioids	112 (13.1)	852	38 (11.9)	318
Anti‐cancer agents	53 (6.2)	852	13 (4.1)	318
HN treatment, *n* (%)				
Isotonic i.v. fluids	378 (44.4)	852	171 (53.8)	318
Hypertonic i.v. fluids	81 (9.5)	852	54 (17)	318
Tolvaptan	38 (4.5)	852	13 (4.1)	318
Fluid restriction	131 (15.4)	852	37 (11.6)	318
Loop diuretic	129 (15.1)	852	29 (9.1)	318
Drug withdrawal	256 (30)	852	98 (30.8)	318
No treatment	53 (6.2)	852	9 (2.8)	318
Laboratory results, median (IQR)				
Plasma [Na^+^], mmol/L	122 (119, 124)	852	120 (116, 123)	318
Plasma [K^+^], mmol/L	4.2 (3.6, 4.8)	852	4.0 (3.5, 4.7)	317
Plasma [Cl^−^], mmol/L	89 (84, 93)	755	88 (82.5, 93)	291
Plasma phosphate, mmol/L	1.02 (0.85, 1.34)	840	0.99 (0.81, 1.34)	313
Blood glucose, mg/dL	120 (102, 151)	852	122 (102, 154)	318
Plasma creatinine, mg/dL	1.0 (0.7, 1.8)	852	0.9 (0.7, 1.6)	318
Plasma urea, mmol/L	6.3 (3.8, 13.8)	852	5.8 (3.7, 13.7)	318
Plasma uric acid, mg/dL	5.3 (3.5, 8.2)	801	5.2 (3.4, 8.2)	296
TSH, mU/L	1.6 (0.94, 2.88)	847	1.56 (0.93, 2.76)	317
Hemoglobin, g/dL	11.9 (10.1, 13.2)	851	12.1 (10.3, 13.5)	318
Serum osmolality, mOsm/kg H_2_O	262 (253, 274)	420	262 (251, 272)	164
Urinary [Na^+^], mmol/L	44 (26, 67)	392	46 (29, 71)	159
Urinary osmolality, mOsm/kg H_2_O	296 (235, 424)	380	310 (237, 440)	154

*Note:* For additional information on individual sodium levels, see Figures S1 and S2.

Abbreviations: ACEi, angiotensin‐converting enzyme inhibitor; ARB, angiotensin receptor blocker; bpm, beats per minute; HN, hyponatremia; ICU, intensive care unit; IQR, interquartile range; MAP, mean arterial pressure; NSAID, non‐steroidal anti‐inflammatory drug; PPI, proton pump inhibitor; SIAD, syndrome of inappropriate diuresis; TSH, thyroid‐stimulating hormone.

^a^ Water intoxication was assumed if urine osmolality was around or < 100 mOsm/kg. Possible causes include psychogenic polydipsia, low solute‐high fluid intake (i.e., “tea & toast”‐diet), beer potomania, etc. Seldom, a low urinary osmolality can also be detected in cases of hypovolemic hyponatremia if the urine sample is obtained after administration of isotonic fluid.

### 3.2 Main Results

#### 3.2.1 Hyponatremia‐Associated Cerebral Edema

Emergency imaging studies were performed in 199 patients; 132 of these patients presented with severe symptoms. In total, six patients presented with global cerebral edema, five of those in the group with severe symptoms (Table 2). Other focal or global lesions were found in three of these patients. Thus, pure hyponatremia‐associated cerebral edema was confirmed in three patients. All three cases were found exclusively in the patient group that had presented with coma as the predominant symptom. Of note, imaging studies revealed other pathological findings in a total of 50 patients (37.9%) of the *severe symptom group* (hemorrhage [27], tumor [8], ischemia [2], hydrocephalus [1], other [12]) and in 24 (35.8%) of the *no severe symptom group* (hemorrhage [6], tumor [5], ischemia [5], abscess [1], other [7]) (Figure 2B).

**TABLE 2:** Clinical characteristics of hyponatremic patients with global cerebral edema on emergency CT or MRI.

Clinical presentation	Sex	Age (years)	Severe symptoms	3% saline	Herniation	Etiology of hyponatremia	Clinical course	LOS (d)	Hyponatremia‐induced edema
1. Found unconscious, vomited, CPR [Na^+^] 119 mmol/L, no known psychiatric history	f	74	Yes	Yes	No	Not known	Died	10	Yes
2. Sudden unconsciousness after complaining about not feeling well, documented drop of [Na^+^] from 142 to 112 mmol/L in 10 h. History of depression and anxiety disorder.	f	38	Yes	Yes	Yes	SIAD due to recently prescribed antidepressant	Died	1	Yes
3. Seizure followed by coma. Had been partying for 2 days, ingestion of high‐volume fluids (urinary osmolality: 58 mOsm/kg). [Na^+^] 111 mmol/L	f	28	Yes	Yes	No	MDMA, excessive water drinking	Survived	17	Yes
4. Initial obtundation, rapid deterioration → coma [Na^+^] 125 mmol/L	m	53	Yes	No	No	Hemorrhage (SAH), global ischemia	Died	3	No
5. Found unconscious [Na^+^] 120 mmol/L	f	82	Yes	No	Yes	Hemorrhage (SAH, ICH)	Died	1	No
6. High altitude fall [Na^+^] 125 mmol/L	f	45	No	No	Yes	Hemorrhage (SAH, SDB, ICH)	Survived	33	No

*Note:* Hyponatremia‐induced cerebral edema was confirmed in patients 1–3. Patients 4–6 had severe intracranial hemorrhage; thus, hyponatremia is likely not the solitary cause of edema. Autopsies were not performed in any of the deceased patients.

Abbreviations: CPR, cardio‐pulmonary resuscitation; f, female; HN, hyponatremia; ICH, intracerebral hemorrhage; LOS, length of stay; m, male; MDMA, 3,4‐methylendioxymethylamphetamine; SAH, subarachnoid hemorrhage; SDH, subdural hemorrhage; SIAD, syndrome of inappropriate antidiuresis.

No emergency imaging studies were performed in 186 patients presenting with severe symptoms. Of these patients, thorough individual evaluation of all available information identified only one patient who was highly likely to have suffered from acute hyponatremia‐induced cerebral edema: a 69‐year‐old woman with bacterial meningitis, [Na^+^] 123 mmol/L, and global edema on delayed CT‐scan (i.e., after transfer to ICU). None of the 45 patients without severe symptoms who died and had not received emergency imaging showed clinical signs suggestive of brain edema.

Taken together, confirmed hyponatremia‐induced cerebral edema was found in four patients, which represents 0.5% of the primary analysis group and 1.3% of the patients with severe symptoms.

#### 3.2.2 Osmotic Demyelination Syndrome

Symptoms suggestive of ODS that developed during the hospital stay (referred to as the possible ODS group) were detected in the medical records of 28 patients (3.3%) of the primary analysis group. Imaging studies were ordered in 19 of these patients (12 × MRI, 7 × CT), which led to the identification of 11 patients (1.3%) with confirmed ODS (Table 3).

**TABLE 3:** Clinical characteristics of patients that developed confirmed ODS during hospitalization.

Nr.	Age (years)	Sex	[Na^+^] on admission (mmol/L)	Δ[Na^+^] at 24 h (mmol/L)	Severe symptoms	Overly rapid correction at 24 h^a^	Etiology of hyponatremia	Pontine/extrapontine demyelination	Risk factors for ODS			
									Malnutrition	Alcoholism	Liver disease	[K^+^] ≤ 3.5 mmol/L
1	49	m	113	10.6	Yes	Yes	Hypovolemia	p + e	No	Yes	Yes	Yes
2	59	m	109	11.0	Yes	Yes	Hypervolemia	p + e	No	Yes	Yes	No
3	65	m	109	10.4	No	Yes	Hypovolemia	p	Yes	Yes	No	No
4	68	f	120	4.2	Yes	No	Hypovolemia	p + e	No	No	No	Yes
5	64	f	123	4.3	Yes	No	SIAD (cause undetermined)	p	No	Yes	No	Yes
6	62	m	121	3.6	No	No	SIAD (COPD)	p	No	No	No	No
7	61	f	113	4.7	No	No	SIAD (drug‐induced)	p + e	No	No	Yes	Yes
8	82	m	109	16.9	No	Yes	Hypovolemia	p	No	No	No	No
9	71	f	106	14.2	Yes	Yes	TAH	e	No	No	No	No
10^b^	28	f	111	25.0	Yes	Yes	MDMA, excessive water drinking	p + e	No	No	No	No
11	65	f	113	10.1	No	Yes	“Tea & toast”‐diet	p + e	No	No	No	Yes

*Note:* Additional information is shown in Table S1.

Abbreviations: COPD, chronic obstructive pulmonary disease; f, female; m, male; MDMA, 3,4‐methylendioxymethylamphetamine; ODS, osmotic demyelination syndrome; SIAD, syndrome of inappropriate antidiuresis; TAH, thiazide‐induced hyponatremia.

^a^ Overly rapid correction at 24 h: [Na^+^] increase at 24 h from baseline > 8 mmol/L.

^b^ This patient is identical to patient #3 in Table 2.

In the subgroup of patients presenting with severe symptoms (*n* = 318), six patients (1.9%) with ODS were identified (among them four with seizures and/or coma). In four of these six patients, emergency imaging studies had been performed revealing one case of global cerebral edema. Of all 28 patients with confirmed or possible ODS, 11 had also received emergency imaging studies on admission. Only one patient (#4 in Table S1) showed signs of demyelination at that early stage which, however, were markedly more prominent in the MRI at Day 20.

Symptoms related to ODS resolved or improved substantially in all cases; no deaths or debilitating sequelae attributable to ODS were recorded.

#### 3.2.3 Risk Factors for Development of ODS

Seven of the 11 patients with confirmed ODS had well‐established risk factors for ODS, and overly rapid sodium correction at 24 h was also confirmed in seven patients. Of All patients with a 24 h‐correction rate > 8 mmol/L, seven patients (2.1%) developed confirmed ODS (7 [2.9%] for a correction rate > 10 mmol/L), and nine patients (2.6%) developed possible ODS (7 [2.9%] for a correction rate > 10 mmol/L). Since ODS is considered to be more frequent with lower initial sodium levels, we also looked at the lower 10% percentile (sodium ≤ 113 mmol/L). In this subgroup, six patients (9.7%) with a correction rate > 8 mmol/L (6 [13.0%] for a correction rate > 10 mmol/L) had confirmed ODS, and two patients (3.2%) had possible ODS (2 [4.3%]).

Due to the low ODS rate, we applied Firth's penalized logistic regression to evaluate the impact of clinical features on risk for possible ODS (for this purpose, we used the composite group of confirmed and possible ODS because of the higher event rate). The independent variables comprised initial sodium, the composite risk factor: alcoholism, liver disease, malnutrition, or hypokalemia [2, 25], and overly rapid sodium correction at 24 h. In the primary analysis group, the composite risk factor, but neither baseline sodium nor overly rapid correction at 24 h, showed a significant influence (model 1). However, in the sensitivity analysis using a reduced dataset with a more reliable estimate of 24 h sodium (model 2), overly rapid correction at 24 h was revealed the most important risk factor for ODS (*p* = 0.004) (Table 4).

**TABLE 4:** Firth's penalized logistic regression analysis with confirmed or possible ODS as the dependent variable.

	Model 1 (*n* = 852, 28 ODS cases)				Model 2 (*n* = 475, 21 ODS cases)			
	Univariable		Multivariable		Univariable		Multivariable	
	OR (95% CI)	*p*	OR (95% CI)	*p*	OR (95% CI)	*p*	OR (95% CI)	*p*
[Na^+^] on admission (mmol/L)	0.91 (0.86, 0.98)	0.009	0.94 (0.88, 1.00)	0.062	0.91 (0.85, 0.98)	0.011	0.96 (0.89, 1.04)	0.289
Other risk factors	2.64 (1.25, 5.79)	0.011	2.43 (1.14, 5.37)	0.021	2.60 (1.10, 6.52)	0.03	2.48 (1.02, 6.32)	0.045
Overly rapid correction at 24 h^a^	2.01 (0.96, 4.35)	0.064	1.65 (0.74, 3.69)	0.219	4.84 (1.92, 14.25)	< 0.001	4.20 (1.55, 12.92)	0.004

*Note:* Multivariable models included plasma sodium on admission (i.e., baseline), other risk factors comprising the presence of at least one of the following: alcoholism, liver disease, malnutrition, plasma potassium on admission ≤ 3.5 mmol/L, and overly rapid correction at 24 h. Model 1 was fitted for the entire primary analysis group; model 2 was fitted for the reduced dataset in which the plasma sodium at 24 h was extrapolated only in patients with available laboratory readings between 18 h and 30 h (yielding a more reliable estimate of the true 24 h sodium). Odds ratio (OR) given per unit change for numeric variables.

Abbreviation: CI, confidence interval.

^a^ Overly‐rapid correction at 24 h: [Na^+^] increase at 24 h from baseline > 8 mmol/L.

#### 3.2.4 Influence of Overly Rapid Sodium Correction on Mortality and Length of Stay

Logistic regression models using either sodium increment over 24 h as a numeric variable (not shown) or categorized rise in sodium (by < 6, 6–10, or > 10 mmol/L in accordance with published work [11]) revealed that mortality was not influenced by the rate of sodium change (Table 5). Likewise, overly rapid correction did not have any impact on the length of stay in our cohort (linear regression, Table S1).

**TABLE 5:** Logistic regression analysis with mortality in the index hospitalization as the dependent variable.

	Univariable model		Multivariable model	
	OR (95% CI)	*p*	OR (95% CI)	*p*
Age (years)	0.98 (0.97, 1.00)	0.01	0.98 (0.97, 1.00)	0.02
Female	0.70 (0.44, 1.11)	0.13	0.78 (0.49, 1.24)	0.29
[Na^+^] on admission (mmol/L)	1.04 (0.99, 1.10)	0.14	1.03 (0.98, 1.09)	0.30
[Na^+^] correction rate 6–10 mmol/L/24 h	Reference		Reference	
[Na^+^] correction rate < 6 mmol/L/24 h	0.95 (0.56, 1.65)	0.84	0.89 (0.51, 1.57)	0.67
[Na^+^] correction rate > 10 mmol/L/24 h	0.66 (0.33, 1.30)	0.23	0.66 (0.33, 1.30)	0.24

*Note:* Odds ratio (OR) given per unit change for numeric variables.

## 4 Discussion

In this analysis of 852 patients presenting to the ED with plasma sodium ≤ 125 mmol/L, only four patients with hyponatremia‐induced cerebral edema were identified. Thus, with 0.5% and 1.3% in the primary analysis group and the subgroup with severe symptoms, respectively, hyponatremia‐induced cerebral edema is a rare event. With 11 patients (1.3%) in the primary analysis group and six patients (1.9%) in the severe symptom group, the event rates for developing confirmed ODS during hospitalization were also quite low but apparently higher than the incidence of cerebral edema.

Hyponatremia‐induced cerebral edema was detected exclusively in patients with coma. It is a widely accepted perception that severe neurological symptoms in the context of low plasma sodium levels can herald life‐threatening complications and should therefore prompt urgent treatment with the goal of elevating the sodium level by 4–6 mmol/L [20]. Although severe symptoms can be observed in chronic hyponatremia, they are believed to be far more common in acute hyponatremia, representing brain swelling [26, 27, 28]. However, our analysis strongly suggests that severe symptoms do not necessarily indicate cerebral edema but more often represent cases of chronic hyponatremia in which adaptive measures have already led to normalization of brain volume [29]. In this situation, the imminent risk of dying is small and may warrant a more informed therapeutic approach.

Withholding immediate therapy has two decisive advantages. First, it enables proper diagnostic work‐up of the underlying etiology of hyponatremia and initiation of a cause‐specific rather than “symptomatic” treatment. Second, the rapidity of sodium rise may be controlled more easily. There is a long‐lasting debate on whether overly rapid correction of hyponatremia is a risk factor for the development of ODS, and recent papers have questioned the need for putting brakes on sodium increase recommended by guidelines [10, 11, 12, 23]. In our analysis, we found 11 cases of ODS, six of them presented initially with severe symptoms. The presence of alcoholism, liver disease, malnutrition, or hypokalemia was confirmed as a risk factor for the development of ODS, in line with published work [2, 30]. Overly rapid correction at 24 h, on the other side, was not associated with ODS risk in the primary analysis group. Similar findings have been published by McMillan et al. [10] and Seethapathy et al. [11]. However, our sensitivity analysis using only patients with a more reliable estimate for the 24 h‐sodium showed that overly rapid sodium correction at 24 h did have a highly significant influence and in fact was the most important risk factor for ODS development. Hence, there might be a considerable bias associated with the methodology of extrapolation of the 24 h‐sodium value which is needed to identify overly rapid correction accurately. If overly rapid correction was present, the risk of confirmed ODS was 2.6% in the overall cohort but 9.7% in those sodium ≤ 113 mmol/L (lower 10%–percentile), underscoring the importance of lower baseline sodium [30].

Seethapathy et al. also reported that limiting the correction rate to < 6 mmol/L/24 h was associated with a higher in‐hospital mortality and longer length of stay, while overly rapid sodium correction (i.e., > 10 mmol/L/24 h) had beneficial effects with regard to these outcome measures. This led to the conclusion that an accelerated sodium‐raising strategy might not only be safe with respect to ODS development but even advantageous. A similar inference was provided by a recent meta‐analysis compiling data from 16 cohort studies (including the trials mentioned above) with almost 12,000 patients [12]; the overall certainty of evidence was ranked low to moderate. In our study, the association of reduced in‐hospital mortality and shorter hospital stay with higher sodium correction rates was clearly not observed. Many reasons (varying designs, settings, correction thresholds, etc.) might be responsible for these discrepancies. We only looked at ED patients and only analyzed the first 24 h after admission rather than multiple periods of 24 h as was done in some of the other studies. Correction rates at Day 1 are probably influenced mainly by therapeutic effort, while at later time points, a higher increase in sodium might merely reflect a more benign hyponatremia phenotype, which then translates into a lower mortality.

In light of the mentioned possible bias generated by the differing reliability of the extrapolated value for sodium at 24 h and the equivocal findings with regard to mortality and length of stay, we are reluctant to abandon the currently advised goals and limits for therapy. As a matter of fact, limiting the rapidity of sodium rise is the only one of several possible risk factors that can be actively controlled.

To our knowledge, this study is the first systematic analysis of the incidence of cerebral edema conducted in ED patients with severe hyponatremia. If the presence or absence of global brain edema differentiates between acute (and life‐threatening) and chronic hyponatremia, it can be inferred from our analysis that chronic hyponatremia accounts for approximately 98% of patients with severe hyponatremia and severe symptoms. If we exclude those 50 patients in whom other cerebral pathologies were found (which may confound our analysis), the proportion of patients with chronic hyponatremia would still be 96%. This is in line with a prospective study in two Swiss hospitals analyzing 298 consecutive ED patients with a plasma sodium < 125 mmol/L, in which 96% of patients were deemed to have chronic hyponatremia solely based on clinical appearance [31].

The high number of almost 40% of patients in the severe symptom group with other focal or global pathologies is remarkable and important for the ED physician to be aware of. The detection of severe hyponatremia should not be considered a sufficient an explanation for severe symptoms per se. In many cases, hyponatremia appears to be a concomitant factor that may contribute to cerebral impairment rather than serving as the primary cause. Other causes must be excluded as they may not only provide an alternative explanation for the presented symptoms but also require urgent treatment. While our findings suggest that it is generally safe to await a full diagnostic workup—including cerebral imaging and laboratory results—there certainly are cases in which immediate treatment is warranted, particularly in the presence of coma or active seizures [32]. Moreover, a bolus of hypertonic saline may also be indicated in hyponatremic patients with space‐occupying intra‐cerebral pathology because even mild superimposed cerebral edema due to hyponatremia may increase intracranial pressure. Therefore, it seems prudent to order emergency imaging studies as an early diagnostic step in severely hyponatremic patients with severe symptoms. Bokemeyer et al. identified focal neurological signs (FNS) as the major predictor for the need for imaging studies with a negative predictive value of 100%, suggesting postponing radiological diagnostics in patients without FNS [33]. This is in contrast to our results. Although we did not explicitly analyze FNS, the presence of severe symptoms seems sufficient to seek clarification by neuroimaging studies.

## 5 Limitations

Both hyponatremia‐induced cerebral edema and ODS are rare events per se, which makes statistically sound analyses notoriously difficult. Given the small sample size and—even more important—the limited number of imaging studies, our single‐center study is underpowered to draw generalizable conclusions. Hence, the true incidences of cerebral edema and ODS are likely to be higher. The lack of imaging studies is of particular importance with regard to ODS detection, especially since our results suggest that this condition might be present more often than commonly believed [30, 34]—also in patients with sodium > 120 mmol/L. It remains unclear whether these patients had a lower sodium before they were admitted to the ED. However, it might also well be that many ODS cases with unspecific symptoms are undiscovered just because ODS is not expected with higher baseline levels. The major strength of our study is the rigor with which the information was collected and scrutinized. A major downside of this study is the uncertainty regarding the true change of sodium at 24 h since timed blood sampling does not apply in this retrospective database, making a mathematical extrapolation necessary. Finally, since we only included patients with initial sodium ≤ 125 mmol/L, no recommendations for patients with mild‐to‐moderate hyponatremia can be deduced from our study. Likewise, while the ECPG recommends a limit of 10 mmol/L rise in sodium in 24 h for patients without additional ODS‐risk factors, we have used 8 mmol/L as cut‐off, which further restricts generalizability.

## 6 Conclusion

Patients presenting to the ED with severe hyponatremia with and without severe symptoms most frequently do not have life‐threatening global cerebral edema and do not necessarily require immediate treatment. The risk of development of ODS seems to outmatch the risk of edema. Overly rapid sodium correction cannot be ruled out as a risk factor for ODS and, thus, recommended treatment limits should be maintained until more evidence is available.

## Author Contributions

V.B. and V.S. conceived and designed the trial. R.R. and J.P.‐M. were responsible for collecting the data. C.H., M.T., and S.C. managed the data and validated the data base entries. V.B., M.J.H., and V.S. scrutinized the identified cases. T.P. and S.H. re‐evaluated all imaging studies. K.M. provided statistical advice, V.B. and V.S. analyzed the data. V.B. drafted the manuscript, and all authors contributed substantially to its revision. V.B. takes responsibility for the paper as a whole.

## Conflicts of Interest

The authors declare no conflicts of interest.

## Supporting information


**Data S1:** acem70158‐sup‐0001‐Supinfo1.docx.

**Data S2:** acem70158‐sup‐0002‐Supinfo2.docx.

**Figure S1:** acem70158‐sup‐0003‐FigureS1.pdf.

**Figure S2:** acem70158‐sup‐0004‐FigureS2.pdf.

**Table S1:** acem70158‐sup‐0005‐TableS1.docx.

**Table S2:** acem70158‐sup‐0006‐TableS2.docx.

## Data Availability

The entire de‐identified dataset will be made available upon request, from the date of article publication by contacting Volker Burst, MD.
