# Supplementary material for: Severe Hyponatremia in the Emergency Department Incidence of Cerebral Edema and Risk of Osmotic Demyelination Syndrome
Source: Acad Emerg Med. 2025 Oct 9;33(1):e70158. doi: 10.1111/acem.70158 (PMC12820600; doi:10.1111/acem.70158)
Supplement: Supplementary file 1 — Data S1: acem70158‐sup‐0001‐Supinfo1.docx. [file ACEM-33-0-s003.docx]

**Data Dictionary Severe Hyponatremia in the ED – *Clinical variables***

*Find patient encounter using the coded study ID

*For all missing data, enter “9999” in SPSS

*Definitions of all variables and the origin of data are listed below

**Demographics*, provided by IT*:**

- Date and exact time of admission in the ED *(date, time)*
- Age in years at time of admission *(numeric)*
- Gender *(male/female)*
- length of hospital stay *(days)*
- patient deceased *(yes/no)*
- transferal to intensive care unit from ED *(yes/no)*

**Laboratory results, *provided by IT (numeric, each value with time of lab entry):***

- Initial plasma sample: sodium, potassium, chloride, phosphate, glucose, creatinine, estimated glomerular filtration rate (eGFR), urea, uric acid, thyroid-stimulating hormone (TSH)
- Initial EDTA sample: hemoglobin, hematocrit (HCT)
- Within 24h of admission: serum osmolality, urine osmolality and urinary sodium
- Throughout hospital stay: any results of plasma sodium or POCT (Blood gas analyzer)

**Vital signs at presentation, *retrieve from ED documentation (numeric):***

- Heart rate (bpm)
- Blood pressure (mmHg)

**Volume status, *retrieve from ED documentation, notes, referral letter; categories:***

- Hypovolemic
- Euvolemic
- Hypervolemic

*🡪 If one of these categories is reported in the ED documentation, transfer to data entry form*

*🡪 If not explicitly mentioned, categorize as follows:*

- - *peripheral edema reported* ***🡪 Hypervolemic***
  - *pulmonary edema reported* ***🡪 Hypervolemic***
  - *ascites reported* ***🡪 Hypervolemic***
  - *exsiccosis reported* ***🡪 Hypovolemic***
  - *volume depletion reported* ***🡪 Hypovolemic***
  - *current or recent episode of diarrhea* ***🡪 Hypovolemic***
  - *no mentioning of any of the above* ***🡪 Euvolemic***

**Etiology of hyponatremia, *retrieve from ED documentation; categories:***

- Syndrome of inappropriate antidiuresis (SIAD)
- Hypovolemic hyponatremia
- Hypervolemic hyponatremia
- Thiazide-associated hyponatremia
- Kidney disease-associated hyponatremia
- Water intoxication

🡪 *if etiology is specified in ED documentation* 🡪 *transfer to data entry form*

🡪 *if etiology is not specified in ED documentation, categorize following these steps:*

- - *urine osmolality <100mOsm/L 🡪* ***water intoxication***
  - *volume status: hypervolemic 🡪* ***hypervolemic hyponatremia***
  - *volume status: hypovolemic or euvolemic and*
    - *urine sodium ≤30 mmol/L* 🡪 ***hypovolemic hyponatremia***
    - *urine sodium >30 mmol/L + thiazide 🡪* ***thiazide-associated* *hyponatremia***
    - *urine sodium >30 mmol/L, no thiazide🡪* ***SIAD***
    - *no urine sodium available, volume depletion explicitly mentioned in ED documentation
      🡪* ***hypovolemic hyponatremia*** *🡪* *if thiazide in medication 🡪 discuss with residents*
    - *no urine sodium available, volume depletion not documented in ED 🡪* ***SIAD*** *🡪* *if thiazide in medication 🡪 discuss with residents*
  - *check eGFR* 🡪 *if <15ml/min* 🡪 *discuss with residents*

**Symptoms compatible with hyponatremia present at presentation or within 24h prior to presentation to ED, *retrieve from ED documentation; categories:***

- Severe symptoms
- Moderate symptoms

*Severe symptoms:*

- coma (Glasgow Coma Scale ≤8, if GCS not available, look for “coma”, “unconsciousness” 🡪 if reported 🡪 coma=yes)
- seizures
- obtundation:
  - somnolence
  - altered/impaired consciousness
  - altered/impaired alertness
  - altered/impaired awareness
  - disorientation
  - confusion
- vomiting

*Moderate symptoms:*

- gait instability
- headache
- nausea
- mood instability
- mild cognitive impairment
- dizziness

**Home medication frequently associated with the development of hyponatremia, *retrieve from ED documentation (yes/no):***

- Non-steroidal anti-inflammatory drugs (NSAID)
- Proton pump inhibitors (PPI)
- Antipsychotic agents
- Anticonvulsant agents
- Antidepressant agents
- Angiotensin-converting enzyme inhibitors (ACEi) / angiotensin receptor blockers (ARB)
- Loop diuretics
- Thiazides
- Mineralocorticoid receptor antagonists (MRA)
- Opioids
- Glucocorticoids
- Anti-cancer agents

**Sodium-increasing treatment in the ED,** **regardless of intent to treat hyponatremia, *retrieve from ED documentation* *(yes/no)***

- isotonic or hypertonic IV fluids (saline, balanced electrolyte solutions)
- tolvaptan
- fluid restriction
- loop diuretic
- withdrawal of home medication frequently associated with the development of hyponatremia (see above)
- other (salt tablets, oral urea, demeclocycline)
- no treatment (= none of the above)

**History of risk factors for ODS *(yes/no)*. *Perform key word search in ED admission report (past medical history, anamnesis). Tick “yes” only if condition is ongoing. In case of doubt, discuss with residents.***

- liver disease (liver cirrhosis, hepatitis)
- history of malnutrition (BMI <18.5kg/m^2^, anorexia, cachexia, weight loss, eating disorder, bulimia)
- history of alcoholism

**Symptoms suggestive of ODS *(yes/no). Perform key word search in medical records (referral letters, notes, specialist consults)***

- Affect lability
- Akinesia
- Apathy
- Ataxia
- Catatonia
- Changes in character
- Cognitive disorder
- Coma
- Concentration disorder
- Confusion
- Delirium
- Dementia
- Demyelination
- Depression
- Dysarthria
- Dyskinesia
- Dysphagia
- Dystonia
- Emotional instability
- Encephalopathy
- Gait disorder
- Hoarseness
- Hyperreflexia, hyporeflexia, loss of reflexes
- Lethargy
- Listlessness
- Locked-in syndrome
- Masseter reflex
- Memory impairment
- Movement disorder
- Mutism
- Myoclonus, opsoclonus
- Ocular motility disorder
- Paralysis
- Pseudobulbar palsy
- Psychosis
- Pupillary motility disorder
- Primitive reflexes
- Reduced vigilance
- Rigor
- Seizure
- Spasticity
- Speech disorder
- Tetraparesis, -plegia
- Tremor
