# Supplementary material for: Severe Hyponatremia in the Emergency Department Incidence of Cerebral Edema and Risk of Osmotic Demyelination Syndrome
Source: Acad Emerg Med. 2025 Oct 9;33(1):e70158. doi: 10.1111/acem.70158 (PMC12820600; doi:10.1111/acem.70158)
Supplement: Supplementary file 2 — Data S2: acem70158‐sup‐0002‐Supinfo2.docx. [file ACEM-33-0-s004.docx]

**Data Dictionary Severe Hyponatremia in the ED - *Imaging variables***

*Find patient encounter using the coded study ID

*For all missing data, enter “9999” in SPSS

*Definitions of all variables and the origin of data are listed below

**Cerebral edema present in emergency imaging (CT, MRI) within 2 hours of hyponatremia detection in untreated patients or patients with ≤2 mmol/L sodium rise, *retrieve from picture archiving and communication system (PACS)* *(yes/no):***

- - Cerebral herniation
    - Subfalcine herniation
    - Transtentorial herniation
    - Central herniation
  - Global cerebral edema
  - Focal cerebral edema
  - Other pathologies (focal or global), categorized:  abscess, hemorrhage, hydrocephalus, ischemia, tumor, other defect

**Signs of osmotic demyelination syndrome (ODS) present in any available imaging studies (CT, MRI) from admission until 3 months after hospital discharge, *retrieve from PACS (yes/no):***

- - Central pontine myelinolysis (with or without extrapontine lesions)
  - Other signs of demyelination
    - Pontine demyelination
    - Extrapontine demyelination
