# Supplementary material for: Severe Hyponatremia in the Emergency Department Incidence of Cerebral Edema and Risk of Osmotic Demyelination Syndrome
Source: Acad Emerg Med. 2025 Oct 9;33(1):e70158. doi: 10.1111/acem.70158 (PMC12820600; doi:10.1111/acem.70158)

Supplemental Figure 1

A. Number of patients in the primary analysis cohort, grouped by initial sodium level and number of patients with severe symptoms (blue)

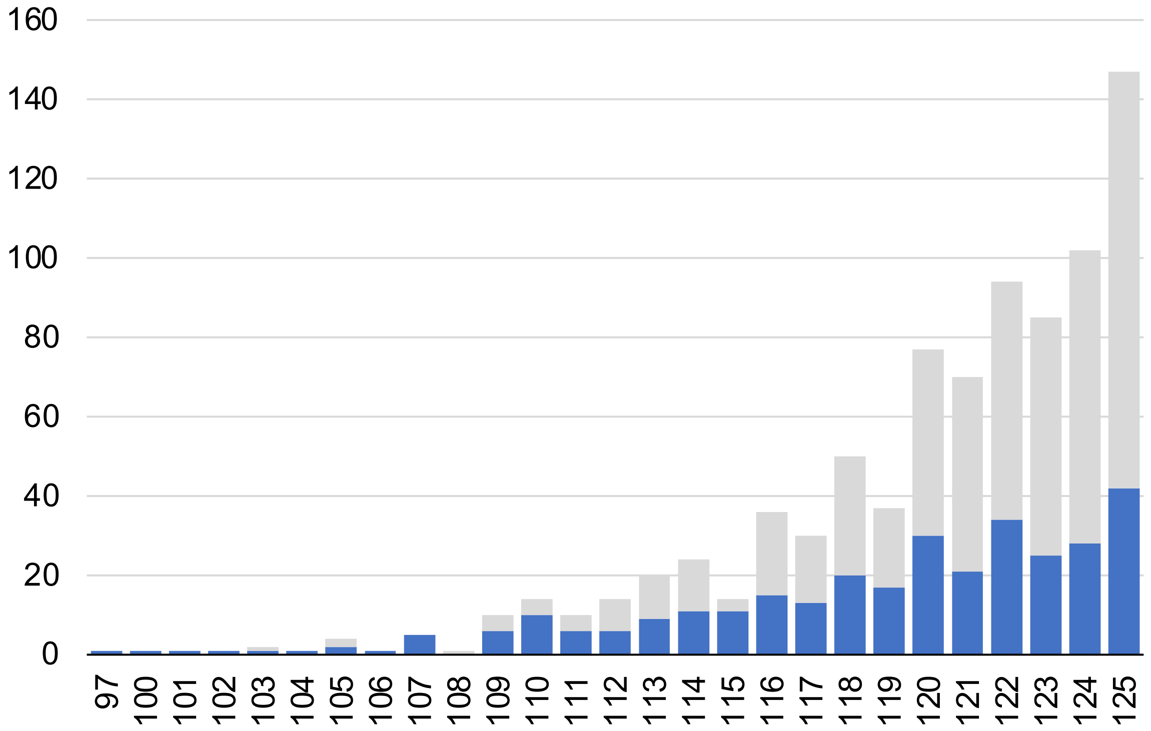

B. Proportion of patients with severe symptoms at each sodium level.

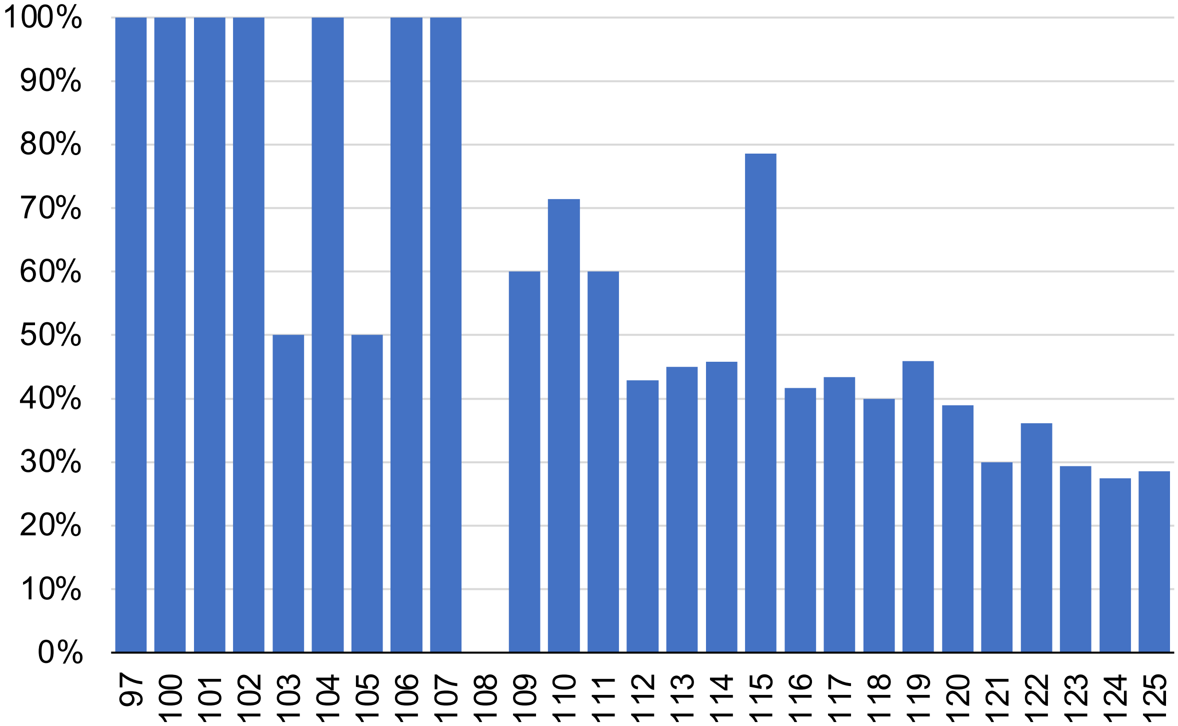

Supplement: Supplementary file 3 — Figure S1: acem70158‐sup‐0003‐FigureS1.pdf. [file ACEM-33-0-s006.pdf]
