# Supplementary material for: Severe Hyponatremia in the Emergency Department Incidence of Cerebral Edema and Risk of Osmotic Demyelination Syndrome
Source: Acad Emerg Med. 2025 Oct 9;33(1):e70158. doi: 10.1111/acem.70158 (PMC12820600; doi:10.1111/acem.70158)

Supplemental Figure 2

A. Number of patients in the primary analysis cohort, grouped by initial sodium level, number of patients with sodium correction rate >10 mmol/L (dark blue) and >8 mmol/L (light blue), numbers on top: confirmed ODS/possible ODS

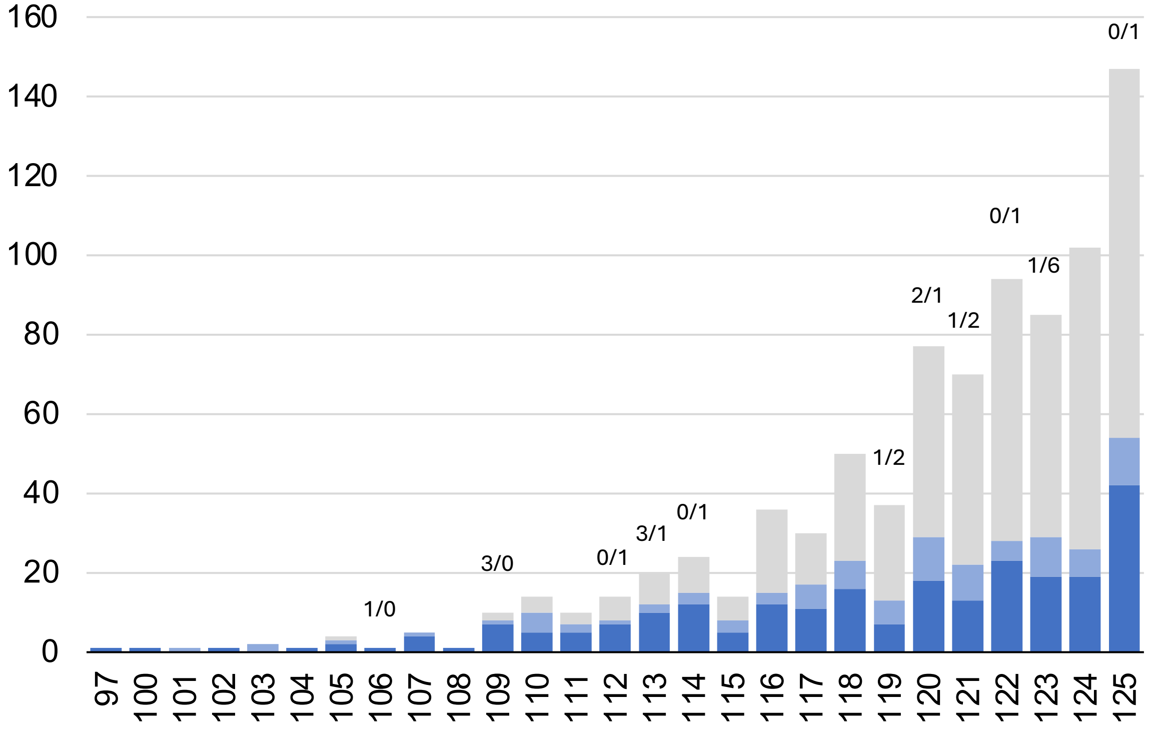

B. Proportion of patients with sodium correction rate >10 mmol/L (dark blue) and >8 mmol/L (light blue)

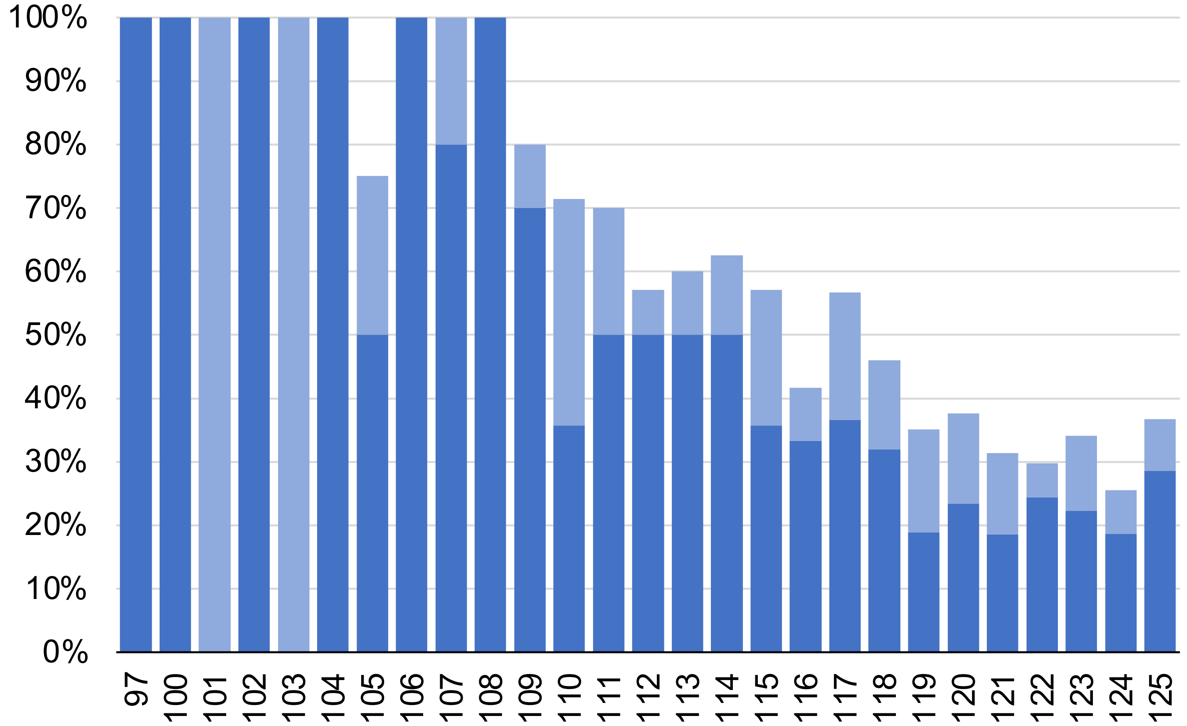

Supplement: Supplementary file 4 — Figure S2: acem70158‐sup‐0004‐FigureS2.pdf. [file ACEM-33-0-s002.pdf]
