# Supplementary material for: Severe Hyponatremia in the Emergency Department Incidence of Cerebral Edema and Risk of Osmotic Demyelination Syndrome
Source: Acad Emerg Med. 2025 Oct 9;33(1):e70158. doi: 10.1111/acem.70158 (PMC12820600; doi:10.1111/acem.70158)
Supplement: Supplementary file 5 — Table S1: acem70158‐sup‐0005‐TableS1.docx. [file ACEM-33-0-s001.docx]

**Supplemental Table 1**

Clinical characteristics of patients that developed ODS during hospitalization. Nr. 1-11: confirmed ODS, Nr. 12-28: possible ODS

| **Nr.** | **Age** (years) | **Sex** | **[Na^+^] on admission** (mmol/L) | **Δ[Na^+^]  at 24h** (mmol/L) | **ODS confirmed or possible** | **Symptoms suggestive  of ODS** | **Onset of symptoms** (in days after admission) | **Time of imaging studies** (in days after admission) | **Course of symptoms** |
| --- | --- | --- | --- | --- | --- | --- | --- | --- | --- |
| 1^#^ | 49 | m | 113 | 10.6 | confirmed | Dysphagia, Dysarthria, somnolence, tetraparesis, tremor right hand | 6 | 10 (MRI) | Symptoms markedly ameliorated until transferal, transferred to rehabilitation after 42 days |
| 2 | 59 | m | 109 | 11.0 | confirmed | Change of personality, somnolence | 4 | 16 (MRI) | Symptoms normalized after 26 days |
| 3 | 65 | m | 109 | 10.4 | confirmed | Somnolence, obtundation, gaze deviation, seizures | 2 | 33 (MRI) | Symptoms markedly ameliorated until transferal, transferred to rehabilitation after 62 days |
| 4^#§^ | 68 | f | 120 | 4.2 | confirmed | Disorientation, slowing, sopor | 2 | 20 (MRI) | Symptoms markedly ameliorated until discharge, discharged after 16 days |
| 5^#^ | 64 | f | 123 | 4.3 | confirmed | Seizure, anisocoria, tremor, dysphagia | 2 | 5 (MRI) | Symptoms normalized after 6 days |
| 6 | 62 | m | 121 | 3.6 | confirmed | Lethargy, dysarthria | 3 | 4 (MRI) | Symptoms normalized until discharge, discharged after 6 days |
| 7 | 61 | f | 113 | 4.7 | confirmed | Dysarthria, ataxia | 2 | 5 (MRI) | Symptoms normalized after 7 days |
| 8 | 82 | m | 109 | 16.9 | confirmed | Confusion, lethargy, complex-focal seizure, | 5 | 22 (MRI) | Symptoms markedly ameliorated until discharge, discharged after 24 days |
| 9^#^ | 71 | f | 106 | 14.2 | confirmed | Spasticity, hypoactive delirium | 6 | 6 (MRI) | Symptoms normalized after 8 days |
| 10^#^ | 28 | f | 111 | 25.0 | confirmed | Delirium, somnolence | 3 | 7 (CT) | Symptoms markedly ameliorated until discharge, discharged after 17 days |
| 11 | 65 | f | 113 | 10.1 | confirmed | Gait disorder, dysarthria, sopor | 3 | 4 (MRI) | Symptoms markedly ameliorated until transferal, transferred to rehabilitation after 9 days |
|  |  |  |  |  |  |  |  |  |  |
| 12^#^ | 56 | f | 122 | 10.2 | possible | Catatonia | Initially sedated. Accurate time point cannot be determined | CT on day12 w/o signs of demyelination | Symptoms moderately ameliorated until transferal, transferred to rehabilitation after 30 days |
| 13 | 77 | m | 113 | 15.7 | possible | Somnolence | 2 | No imaging studies performed | Symptoms normalized after 9 days |
| 14 | 71 | m | 123 | 10.4 | possible | Confusion, dysphagia | 4 | No imaging studies performed | No neurological status documented |
| 15 | 73 | f | 125 | 0.5 | possible | Depression, emotional instability | 8 | No imaging studies performed | Symptoms normalized until discharge, discharged after 32 days |
| 16 | 44 | m | 121 | 17.9 | possible | Dysarthria, dysmetria (ataxia), confusion | 6 | CT on day10 w/o signs of demyelination | Symptoms ameliorated initially, developed septic shock on day 10, died at day 12 |
| 17^#^ | 65 | f | 120 | 17.4 | possible | Delirium | 3 | CT on day 4 w/o signs of demyelination | Symptoms normalized until transferal, transferred to rehabilitation after 6 days |
| 18^#^ | 61 | m | 121 | 3.3 | possible | Ocular motility disorder, confusion | 2 | MRI on day 3 w/o signs of demyelination | Symptoms normalized after 6 days |
| 19 | 80 | m | 123 | 2.6 | possible | Lethargy, confusion | 3 | No imaging studies performed | Symptoms normalized after 8 days |
| 20^#^ | 55 | m | 112 | 11.8 | possible | Delirium, gait disorder, ataxia | 3 | No imaging studies performed | Symptoms ameliorated until transferal, transferred to rehabilitation after 6 days |
| 21 | 50 | m | 123 | 9.4 | possible | Confusion, delirium | 4 | MRI on day 4 w/o signs of demyelination | Symptoms normalized after 11 days |
| 22 | 59 | m | 123 | -1.4 | possible | Changes in character (aggressiveness), cognitive disorder | 2 | No imaging studies performed | Symptoms normalized until discharge, discharged after 18 days |
| 23^#^ | 56 | f | 119 | 10.5 | possible | Seizures, psychosis | 6 | CT on day 6 w/o signs of demyelination | Symptoms normalized until discharge, discharged after 12 days |
| 24 | 58 | m | 123 | 6.7 | possible | Aphasia, confusion | 2 | CT on day 6 w/o signs of demyelination | Symptoms ameliorated until discharge, discharged after 21 days, but persisting aphasia |
| 25 | 70 | m | 119 | 9.7 | possible | Confusion, delirium | 2 | No imaging studies performed | Symptoms normalized until discharge, discharged after 6 days |
| 26 | 82 | f | 114 | 0 | possible | Gait disorder, somnolence | 3 | CT on day 4 w/o signs of demyelination | Symptoms normalized until discharge, discharged after 14 days |
| 27^#^ | 81 | f | 119 | 3.2 | possible | Seizures, reduced vigilance | 2 | No imaging studies performed | Symptoms normalized until discharge, discharged after 6 days |
| 28 | 59 | m | 123 | 1.5 | possible | Cognitive disorder, apathy | 2 | No imaging studies performed | Symptoms normalized until discharge, discharged after 7 days |

^#^patients in whom emergency imaging studies had been performed

^§^patients who showed signs of demyelination already in emergency imaging study
