# Supplementary material for: Severe Hyponatremia in the Emergency Department Incidence of Cerebral Edema and Risk of Osmotic Demyelination Syndrome
Source: Acad Emerg Med. 2025 Oct 9;33(1):e70158. doi: 10.1111/acem.70158 (PMC12820600; doi:10.1111/acem.70158)
Supplement: Supplementary file 6 — Table S2: acem70158‐sup‐0006‐TableS2.docx. [file ACEM-33-0-s005.docx]

**Supplemental Table 2**

Linear regression model with length of stay (in days) as dependent variable.

**Univariable models**

|  | **b** | **Std. Error** | **p** |
| --- | --- | --- | --- |
| Intercept | 17.36 | 2.60 | <0.01 |
| **Age** (years) | -0.09 | 0.04 | 0.02 |

|  | **b** | **Std. Error** | **p** |
| --- | --- | --- | --- |
| Intercept | 11.73 | 0.80 | <0.01 |
| **Female** | -0.83 | 1.12 | 0.46 |

|  | **b** | **Std. Error** | **p** |
| --- | --- | --- | --- |
| Intercept | 17.68 | 14.31 | 0.22 |
| **[Na^+^]** **on admission** (mmol/L) | -0.05 | 0.12 | 0.66 |

|  | **b** | **Std. Error** | **p** |
| --- | --- | --- | --- |
| Intercept | 11.51 | 1.14 | <0.01 |
| **[Na^+^] correction rate 6-10mmol/L/24h** | Reference |  |  |
| [Na^+^] correction rate <6mmol/L/24h | 0.41 | 1.39 | 0.77 |
| [Na^+^] correction rate >10mmol/L/24h | -1.50 | 1.59 | 0.34 |

**Multivariable model**

|  | **b** | **Std. Error** | **p** |
| --- | --- | --- | --- |
| Intercept | 34.12 | 15.38 | 0.03 |
| **Age** (years) | -0.10 | 0.04 | 0.02 |
| **Female** | -0.45 | 1.14 | 0.70 |
| **[Na^+^]** **on admission** (mmol/L) | -0.13 | 0.13 | 0.28 |
| **[Na^+^] correction rate 6-10mmol/L/24h** | Reference |  |  |
| [Na^+^] correction rate <6mmol/L/24h | 0.70 | 1.41 | 0.62 |
| [Na^+^] correction rate >10mmol/L/24h | -1.81 | 1.59 | 0.26 |

b**,** regression coefficients

Adjusted R^2^ .003

Both, p-values as well as the low R^2^ indicate lack of a meaningful association between correction rate and length of stay.
